# The Patient’s Attitude Toward Type 2 Diabetes Mellitus, a Qualitative Study

**DOI:** 10.1007/s10943-014-9848-9

**Published:** 2014-03-06

**Authors:** Reyhaneh Abolghasemi, Mojtaba Sedaghat

**Affiliations:** 1 Medicine, Quran and Hadith Research Center, Baqiyatallah University of Medical sciences, Tehran, Iran; 2 Unit609-Block 5, Pezeshkan Complex, Nirougah-Shahran Second Sq, Iranpars St-Poonak Sq, 1478865748 Tehran, Iran; 3 Public Health Department, Tehran University of Medical Sciences, Tehran, Iran

**Keywords:** Diabetes, Phenomenology, Qualitative analysis, Health care, Users’ experiences, Health

## Abstract

Diabetes is an important health problem through the world. In comprehensive diabetes care, therapists must understand not just the observable behavior but the underlying attitudes which drive that behavior. Health, then sickness, has many aspects, and one of the famous descriptions is guided by WHO. This study aimed to explore dimensions of attitudes in diabetic patients about their disease, attending two medical centers in *Tehran* (capital of *Iran*). We conducted the open semi-structured face-to-face interviews with 27 patients. We used new methods for collecting data, reliability, validity, analyzing and ethical approval. We identified eighteen themes in four aspects of attitude: physical, mental, social and spiritual. Based on health promotion idea, we can divide themes in two broad categories: progressive attitude toward the higher level of health care and inhibitors attitude for this. The result of this research can be used in evidence-based education and management programs in comprehensive care of type 2 diabetes mellitus.

## Introduction

The worldwide prevalence of diabetes mellitus (DM) has risen dramatically over two past decades (Braunwald 2005). Prevalence of diabetes in adults was estimated to be 4.0 % in 1,995 and to rise up 5.4 % by the year 2025. The number of adults with diabetes in the word will rise from 135 million in 1995 up to 300 million in 2025. By the year 2025, more than 75 % of people with diabetes will reside in developing countries, compared with 62 % in 1995 (King et al. 1998).

The prevalence in Tehran is 7–8 % (Larijani and Zahedi 2001), meanwhile in people over thirty years old is 7.3 % (Delavari et al. 2004).

Health—then sickness—has many aspects. The most famous description is guided by WHO. It has three kinds: physical, mental and social (World Health Organization 1946). More recently, the spiritual dimension of health was highlighted in the Bangkok charter for health promotion (Hawks et al. 1995). Therefore, some experts have suggested Holistic Wellness Models (The Bangkok charter 2005). Even though others had shown in educational and promotion health programs, it has not been enough planning for all or some of them (Hawks et al. 2008).

In comprehensive diabetes care, patient education and their self-care are very important (Braunwald 2005). Understanding the behavior of persons toward their problems requires knowing of their attitudes about it. Otherwise, for any successful educational and therapeutic program, in meeting a particular community, providers need to be informed by understanding of patient’s attitudes and beliefs.

There are many explanations about attitude. Alport cited sixteen definitions in 1935.

For example: “The attitude is a mental and neural state of readiness, organized through experiences, exerting a directive influence upon the individual’s response to all objects and situations with it is related” (Stonea et al. 2005). In this definition, attitude has a directive and/or dynamic influence on behavior (Alport 1935). Any change in the belief about things causes change in feeling, in exciting to act and, therefore, in behavior.

Review articles have shown that many kinds of study about attitudes in diabetic patients have been down in different countries. In some researches, the attitudes have been examined through quantitative methods. In qualitative methods, it has been either one of the factors (for example nutrition, exercise or spiritual) or some dimensions by different research planning, data collections, analyzing and discussion. Except the Carmen’s (Adams 2003), phenomenology method has rarely been used in the study of diabetes. It has never been studied multidimensional attitude toward health and disease (based on WHO theory).

In *Iran* (in sites: Google-*SID*-*Iranmedex*-*Irandoc*), totally there are twelve research articles about attitude in diabetic patients, and only one of them is qualitative (grounded theory) (Masoudi Alavi et al. 2004) and all others are quantitative (KAP study).

For better evaluation, treatment and promotion of health in such chronic diseases, managers need to understand their patients’ points of view about their sickness. For this reason, we interviewed with diabetic people which had more than 1 year history of diagnosis. We checked all four aspects of health. We have used new planning, methods and analyzing too.

## Methods

### The Strategy (Paradigm)

One of the evaluating strategies in attitude is the qualitative method. A qualitative research is designed to observe social interaction and understand the individual perspectives. Also, it provides insight into what people’s experiences are, why they do, what they do and what they need in order to change.

Strauss and Corbin describe it: “qualitative research is any kind of research that produces findings not arrived at by statistical procedures or other means of quantification” (Streubert and Carpenter 1999).

Some public qualitative strategies are grounded theory, ethnography, phenomenology, historical research and case study.

In this research, our object was exploring the patient’s attitude for applying in educational programs. One of the best methods for this purpose is a phenomenological method. Because the phenomenon is the phenomena at nature, phenomenology is a philosophy method and approach that valued for its focus upon describing respondents “Lived experience” (Elder and Miller 1995). In this kind of study, a researcher through investigating people’s natural history (actions—experiences—sensations), and their tales clarifies meanings of the phenomenon.

Plager (1994) considers Heideggerian phenomenology to be an appropriate methodology, as it helps us to understand activity in the context of everyday life (Patton 1987).

Our method in this research is similar to Heidegger and Gadamer’s Hermeneutic theory; also, there are some differences between our Islamic idea and them.

### The Setting

Two communities for study was in two medical centers in *Tehran* that represented different socioeconomic status; Diabetes clinic in Endocrinology & Metabolism Research Center (*EMRC*) in *Shariati* Hospital with better status and *Aboozar* Clinic, a public outpatient center in south of Tehran with low socioeconomic status.

In qualitative research (unlike a quantitative), researcher is in text of research and had an important role within it. In this study, a researcher has done interviews in two times, first as the university researcher (complete observer), second as a training resident in internal medicine course (observer as participant). So the interviewees’ viewpoints have been considered in both sides. Due to similarities in the culture of researcher and patients, it was easy to have best connection and understand each other.

### Sampling Method

In *Shariati* Hospital, patients diagnosed previously and had follow-up files. But in *Aboozar* Clinic, they visited for different diagnostic and therapeutic reasons.

Those who met inclusion criteria (based on National Diabetes Data Group (*NDDG*) and WHO protocol (Braunwald 2005), and those who had physical and psychological readiness, and after agreement, were interviewed individually. During communication between *Turkish* people, there was a *Persian* translator with them.

The sample size was based on similar studies, but the critical determinant was saturation during the research process (purposeful sampling) and criterion for stopping data collection was information overlapping. Finally, after the interview with twenty-seven patients, we gained saturated information.

### Methods for Collecting Data

Semi-structured with open-ended questions was undertaken with each participant to make sure that four domains in WHO’s definition were covered by all subjects.

At the beginning of speech, we prepared a general question such as “What is Diabetes?” Then there were some questions to cover criteria in attitude, for example: “What is the relation between DM and human body?” (Physical phenomena) “Please describe about Diabetes and mental feeling” (Psychological phenomena). Is there any relationship between community and DM? “(Social phenomena)”. Of course, the sentences were not previously defined, and we try to use their own words. Next questions were done during the interview process.

We allowed the subjects to “tell their story” in “their own words” and in no particular order, but the conversation was kept flowing by Patton’s strategies.

To collect full information, after permission, their talks were recorded by microphone and tape-recorded.

Some handwritten notes in relation to nonverbal communication (researcher’s observations, interpretations, feelings and reflections) were taken during and immediately after the interviews and complemented verbal transcription.

We used a face sheet to record information such as date, time (beginning and end of the interview), place, personal medical history and demographic information about the participants. At the end of an interview session, we asked their information source.

### Analyzing Methods

We transcribed each question and responses from audiotape verbatim and completed by handwritten notes. The interviews were analyzed according to Colaizzi’s method (1978) of phenomenological inquiry. It has seven procedural steps (stages) for analysis: Stages 1 (Protocols) and 2 (Extracting significant statements) were done by recorded tables and notes. We used exactly the same word that they used. In stages 3 and 4 (3: formulating meanings of themes, 4: clusters of themes) we have used Q analysis (Cross 2005) to find out logical points between statements. In addition for increasing accuracy, this method arouses information’s validity because diabetics were sharing in analyze process. The stages 5 (Exhaustive description) and 6 (Fundamental structure) were repeated by peer researchers (three residents) in the department of community medicine in Tehran University of Medical Sciences (TUMS). Overall, 75 % of statements received identical topics and same fundamental structure.

### Reliability and Validity

Although reliability and validity are treated separately in quantitative studies, these terms are not viewed in qualitative. Instead, a terminology that encompasses both, such as credibility, transferability and trustworthiness, is used. Also, the way for achieving validity and reliability of a research get affected from the qualitative researcher’s perspectives. So researcher should eliminate bias and increase the researcher’s truthfulness of a proposition about some social phenomenon.

Authenticity is demonstrated using rich description and by the participant’s words. Using multiple methods and sources of data collection strengthens our claim for fair dealing in illuminating the phenomena using different perspectives.

Triangulation is typically a strategy (test) for improving the validity and reliability of research or findings. Patton (2001) advocated it by combining methods. This mean using several kinds of methods (both quantitative and qualitative approaches) or multiple methods of data collection and data analysis, but does not suggest a specific method for all of researches and depend on their criterion in process.

We collected information through interview and direct observation. For analyzing, we used both qualitative and quantitative style (Colaizzi & Q methods). Informants participated in analyzing process after 3 months. Five patients helped us. By these methods, the observation effect decreased. Using the colleges views increased validity.

The generalizability, as one of criteria for qualitative studies, depends on case selecting and method of study. Because a final object was operational findings, we selected patients from two different socioeconomic areas. Different researcher role was for this reason too. Besides an individual analysis, we did a total analysis by combining interviews. This was difficult and time spending, but increased external validity.

### Ethical Approval

There are some risks for attendance in qualitative research such as anxiety and distress, exploitation, misrepresentation, identification of the participant by self or others and inconvenience and opportunity cost.

To reduce these risks, we have done the following tasks:

Scientific soundness achieved by ethical approval from a local research ethics committee in TUMS and in both health centers. The protection code of human subject for medical research in TUMS (Protection code in URL) is based on international rules (Helsinki and Nuremberg manifesto and Belmont report) and Islamic religion.

Because the researcher was not a manager, so it was not the exploitation in doctor–patient relationship. Their doctor or practice nurse approached patients initially, and then, care-workers ensure them that supportive observations are available whenever it is necessary.

To decrease misrepresentation and misinterpretation, we used different ways to increase validity of findings.

To ensure the patients their information will be hidden, we have never asked them their first and second name, address, national code or any personal indicator.

The written consent was obtained from the participants after they have been verbally agreed. Then, they gave time to consider their participation and ask questions from researcher.

Autonomy (capacity for self-direction, withdraw at any time) and beneficence–non-maleficence–justice completely regarded in all over the interview.

With patients’ participation in analysis process, treating informed consent was an ongoing process rather than a one-off event at the beginning of the study.

Findings:

At first, we did preliminary interview with four patients (two people in every center) to try out protocol. After this, we omitted questions about prevalence of DM in the world and Tuberculosis and Malaria from disease ranking (pile sorting exercise) because patients have not been enough information (and so attitude) toward them.

Twenty-seven of the 30 subjects agreed to be interviewed. The initial interviews had taken within 1 h but later by omitting some questions (after saturation of information); it was decreased.

Table 1 is the demographic character of patients.

**Table 1:** Characteristics of patients interviewed

Property	*N* (%)
Place	
Aboozar Clinic	15 (55.6)
Shariati Hospital	12 (44.4)
Sex	
Female	22 (81.5)
Male	5 (18.5)
Age (years)	
≤20	0 (0)
21–40	2 (7.4)
41–60	18 (66.7)
61–80	5 (18.5)
≥81	2 (7.4)
Employment	
Housewife	22 (81.5)
Employed	1 (3.7)
Worker	2 (7.4)
Retired	2 (7.4)
Education	
Illiterate	6 (22.2)
<6 years school	14 (51.9)
≥6 years school	2 (7.4)
School graduate	4 (14.8)
BS	1 (3.7)
Current treatment	
Oral medication	23 (85.2)
Insulin	4 (14.8)

Mean age of participants was 54 (range 25–86) years, and all of them married. The family history of diabetes mellitus was 60 %. Mean year from diagnosis of DM was six (range 1–17) years. Only one person had classic symptoms (polydipsia and polyuria) at the beginning, and two persons were diagnosed after the other reasons (for example, routine checkup), but most of them said non-specific symptoms (anxiety, headache, pruritus, knee pain, vertigo and so on).

At time of research, many (67 %) of patients suffered from polydipsia, polyphagia and polyuria showing poor control. Nineteen people (70 %) had side effects, and it was predominantly: neuropathy, retinopathy, cardiovascular and nephropathy, respectively. Other medical problems were hypertension and depression. Much less was peptic ulcer, low back pain, arthralgia, varicose veins, breast cancer, hirsutism and Dysuria.

### General Attitude About DM

Their believes were very different, so these themes were gained:

#### Definition of Diabetes

Most of them explain DM as a mysterious internal problem. For example, an 44-year-old woman said: “It is similar a red, beautiful apple but worms are eating internally” and an 85-year-old man said: “It destroyed total of the human body, and so I can’t do my daily works,” but some of them had good views and said pathology or its treat ability: “With suitable food, diabetes fully treated.” “In this disease an organ demolished” (an 50-year-old man).

#### Causes of DM

Patient’s attitudes about causes of DM are shown in Table 2.

**Table 2:** Beliefs about causes of DM and examples

Beliefs	Examples (the patients sentences)
Psychological and emotional related factors	“It’s due to sorrowing And nervousness, so when I’m glad my blood glucose is normal” (an 65-year-old woman)
Life style factors and industrial living	“Low activity is epidemic and rice, bread, sugar and salt is mostly in meal” (an 52-year-old man)
God will	“It’s all to do with God whether you get it (diabetes) or don’t get it” (an 85-year-old man)
Genetic factors	“My dad was sick so am I” (an 45-year-old woman)

Many of them believed that its cause is emotional conflicts. A woman, who has gestational diabetes mentioned that measles, mumps and rubella (MMR) vaccine during her pregnancy, was an etiologic factor. An 44-year-old woman said, “Atmosphere and its pollutant materials cause diabetes.”

#### Importance of DM Compared with Other Diseases (Pile Sorting Exercise)

**Fig. 1:**
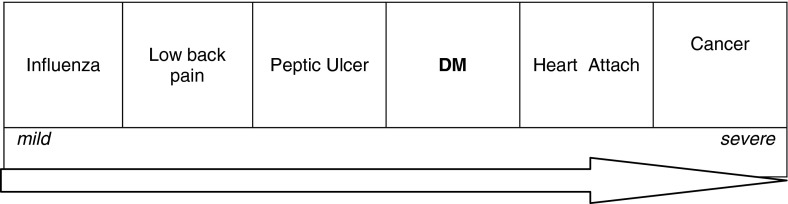
Patients’ attitude about DM relative to other diseases

To know the value of DM in comparing with other diseases, we used innovated method. We asked to arrange six diseases according to priority. As it is shown in a diagram (Fig. 1), the position of DM comparison with five diseases was nearly in middle part. Some examples of participant’s words about their ranking are as follows:

Cancer: “It is corrosive, painful, and fatal and without prevention,” “its cost is high,” “I wish God not will.”

- Heart attack: “It killed or paralyzed suddenly.”
- Peptic ulcer: “It is similar to DM because of the rule of diets in both are the same.”
- Low back pain: “It isn’t diseases; it is due to hard working” “It is cured with resting.”
- Influenza: “It is transient, not chronic” “It has prevention and treatment.”

#### Types of DM

They divided the DM in two categories with different views, but the common belief was based on symptoms “My sister becomes thirsty, but I am hungry” (an 55-year-old woman). “My dad’s foot felt to burn, but my tong is” (an 47-year-old woman). The other criteria were age onset (from childhood or adolescence), disease severity (mild or severe), complications (without side effects or with disabilities complications), treatment (oral hypoglycemic pills or insulin), glucose level (low or high), personal figure (thin or fatty), etiology (heredity or inherited).

#### Prevalence in Sex and Age

The informants believed that DM becomes common after 40 years old. Only two people said that it is common in all over the lifetime. Most of them agree with preference in females, then was equality for both sexes and less attitude was about superiority in men.

#### Physical State

According to patients’ attitude, there are relationship between diabetes and human body, and it has been told five themes as follows:

#### Importance of Diagnosis

They believed on-time diagnosis is very important and causes effective treatment and more living time. An 52-year-old man said: “Late diagnosis causes side effects, disease progression or even early death.” They requested personal testing and examinations, although there are different kinds and frequencies. “I check my blood pressure every two months and 1 year ago I tested urine and blood glucose” (an 83-year-old woman said). The reason was mostly due to lack of sufficient information, expensive cares and social problems such as crowding in clinics and laboratories and traffic jam. “Commuting to a clinic is difficult because of traffic” “After leaving previous doctor, there aren’t periodical examinations” (an 55-year-old woman). Fewer patients had glucometer in their home, and they did not do regular checking.

#### Nutrition

Their believes usually were often based on medical information. Most of them had good appetite on chocolate and confectionary, while they should never use, although there were some attitudes with cultural origin. “Fried vegetables and boiled eggs are suitable. Instead of sugar, they should use sweet fruits such as date and dried rape with tea” (an 60-year-old woman). They mentioned: variation in diet, less quantity with more time-saving during a meal and adjusting to personal nature (four humors based on Iranian traditional medicine) can help to recover nutrient status.

They mentioned that there are some problems for not following true regimen;

Hypoglycemia symptoms, other diseases such as peptic ulcer, bad tasting, and mental disorders.

Based on patients’ speech, the researcher believed that the main reason for not following safe diet program is lacking of high excitement, which is caused by above-mentioned causes.

#### Physical Activity

Some people told about the kind of sport: “Group sports are suitable” (a 37-year-old woman). “Climbing and walking is good” (an 57-year-old woman).

Some of them mentioned about the effect of exercise in decreasing blood glucose or feeling better condition: “When I exercise before breakfast my body will be relaxed” (an 65-year-old woman).

For most of them, the reason of not having sport was personal problems “From 1 year ago I could not exercise due to my knee fracture” (an 70-year-old man), and the predominant cause was psychological: “From 4 years ago my walking has been stopped because of my family problems” (an 56-year-old woman), “If people have desire to exercise it is good” (a 36-year-old woman).

#### Attitude About Body Shape and Weight

The patient’s views were based on medical objects or from social norms. For example, an 44-year-old woman said: “Obese abdomen has bad feature and reducing the weight will reduce diabetes.”

- “This disease looses of weigh and makes figure changing.” (an 64-year-old woman)
- “There is no relationship between diabetes and being fat or slim, because one of my friends is 150 kg weight, while he has no diabetes” (an 58-year-old man).

However, this phrase: “I like to be slim with good figurative” has told by a lot of them which shows importance of the social views which means fatty posture is very shameful. But if we consider their age, culture, social status and business, it does not have effective influence on their attitude and practice. Even though some of them do not accept to be obese, and the others wanted to gain more weigh. The Q analysis shows these two different sights.

#### Side Effects and Complications

They feared chronic (loss of vision, kidney failure, limb amputation) or acute (heart attack, infection) complications. Other problems that they said were liver failure, stomach problem, hypertension, dizziness and depression.

### Mental State

#### Psychological Reaction to Diagnosis

For some people, the diagnosis had been such a shock, and others (younger patients) were comfortable. But some of them accepted their diagnosis with resignation and expressed that God had sent their condition. “It is all to do with God whether you get it [diabetes] or do not get it” (an 85-year-old man). “At first I was worry, but I accepted diabetes as a gift from my God” (an 57-year-old woman).

#### Disease Effect on Mental Health

Some of the interviewees mentioned anxiety and frustration associated with their experience of diabetes: “I do not happy and worry because of my bad disease” (an 64-year-old depressed woman). Their attitude affects other aspects of behavior such as diet and exercise.

#### Attitude to Future

Anxiety was often related to concern about possible future complications of diabetes: “I just got worried; despite this I wish God shall be not blind, and my foot will not be ulcerous” (an 56-year-old woman). “I don’t know what is going to happen in time ahead” (an 50-year-old man). Some of them were a little worry about their parents and children. On the other hand, patients in particular, appeared to find coping with important health difficult: “I am resigned and satisfied to God’s will” (a 37-year-old woman).

### Social State

#### Prevalence in Iran and Its Cause’s

Most of them believed that it has become more common comparing to past years. They said two broad categories of causes for this excess:

- Psychological factors: stress, sorrowing and nervousness.
- Diet-related factors: Manufactured foods, over feeding, fats and rice.

#### Patients’ Beliefs About Social Constraints Resulting From Diabetes

It seems that there are a range of social attitudes; from unconscious (mostly in non-diabetic persons) to perceive completely “You must do restrictions in your diet” (a boy said to her mother). Some people had pity feeling: “Poor patient” or even fearing “Diabetes is contagious.” One interviewee raised possible negative effects of sharing experiences with peers in the absence of good understanding of diabetes, in terms of potential increasing in tension and anxiety: “I am comfortable if I do not keep talking about it” (a 27-year-old young woman).

#### The Role of Family Member

There were three important aspects which they should be done by their home families:

- Emotional support: “If there wasn’t my husband’s support, I would feel sadness” (a 27-year-old woman).
- Enough information: “We must notice that diabetes is not contagious and harm full to the others” (an 44-year-old woman).
- Current medical behavior: “They should not prepare prohibited food for example, rice and fried foods” (an 60-year-old man).

They confirmed the role of mass media in public information. These days some programs have became much better than before, while there are not enough force to obey safe food recipe: “They think they are allowed to use confectionary as much as they want” (an 58-year-old man).

### Spiritual State

In Iran religious society, Muslim patients’ aspects could easily affect their talking. So we explain two relative believes:

#### Religious Attitudes Cause Disease

They referred to God as existence of everything, including diseases: “Fait is on God forth and to become a sick, is for examination of the creatures.” “We will be sick to call God for our health” (a 38-year-old woman).

#### Religious Attitude Toward Treatment

“I should obey safe diet by my God’s help.” “All health is on his hand (God), and I will gain my health only by All-might God” (an 70-year-old man).

## Discussion

In this research, we have observed diabetes attitudes about type 2 DM by phenomenology qualitative research. The main idea was WHO’s definition about health. We gained 13 themes. In each item, there are multiple aspects and each belief has different results. We believe that any people should continue their improvement and progress, so every day and minute he/she should go ahead.

We can divide these results of themes into two broad categories:

- Progress attitude toward the highest degree
- Inhibited attitude to be progressed

Of course relating each special attitude to these categories depends on personal condition, and sometimes it is difficult. For example, if attitude about prevalence of DM in existing society deals to a normal condition and is accepted, it will be an inhibited belief. Otherwise, if it is seen as a big problem, it will be a progress attitude. The religious beliefs convicted that God gives fate, so anxiety and depression after diagnosis decreased. Allah has sent diseases and treatments, and a monotheist person must struggle for best care and living with full health. Such views are usually held in parallel with acceptance of individual responsibility and potential for change. Similar to this opinion, mentioned by Masoudi Alavi et al. (2004), Stonea et al. (2005), Leidy and Hasse (1999) and Greenhalgh et al. (1998). On the other hand, if the result of this idea is carelessness or laziness in obeying the medical instructions, these attitudes will have no positive effects. Other observers in their studies have referred to these points.

In the only one qualitative research that was done in Iran (based on grounded theory) (Masoudi Alavi et al. 2004), three original systems were extracted through fundamental theory (personal system care, cure and support system and social system). Other researches are quantitative.

Based on our interviewees believes the main cause of disease was emotional. In New England’s women, stress was one of the diabetes’ reasons and itself was the result of diabetes (Vader 2006). We could find this emotional attitude, but “profound sadness” scarcely could be found in our patients.

The state of DM comparison with other diseases (Fig. 1) may be due to mortality of them. Although this sorting is relatively corrected, but it is not documented, then it should not bring carelessness or fearful in DM management process. Believing about more prevalence in older ages and women may cause inattention behaviors (in younger or men) people in diet and activity.

Modifying diet and dizziness or weakness related to it was similar to Stonea et al. (2005) and Russell’s (2001). In Persel (2006) research woman’s attitude toward nutrition—“Loss of eating and drinking pleasure”—was similar to ours.

In our research, personal (physical or mental) was the most reason for living sports, but in the Carter Nolan et al. (1996) study, it was cultural reasons (transportation, childcare and an exercise environment).

Most of our patients worried about chronic complications, similar to Kapur et al. (1997), Gillibrand and Flynn (2001) and Smith et al. (2003) research, but in Greenhalgh et al. (1998) fears from acute morbidities was dominant.

Many of participants in this research had accepted their diagnosis with minor stress, and there was not any devastating condition opposite to patient’s expression in Greenhalgh et al. (1998) and Heuer and Lausch (2006) study.

Health-related anxiety and sometimes depression were commonly suggested in those interviewed, particularly in Aboozar Clinic, but patients with family history of diabetes experienced lesser anxiety. Strong family relations play an important role in side of attitude and experience.

Linkage between anxiety, depression and diabetes needs further clarification, but a meta-analysis showed that the risk of depression in controlled studies of patients with type 1 and type 2 diabetes was twice that of the non-diabetic comparison group (Anderson et al. 2001).

In this study, similar to Miglani et al. (2000) some interviewees said that they did not want to discuss their disease with their friends and acquaintances because people would treat them differently or perceive them as sick. The research that Lopez et al. (2005) had done by a qualitative (ethnographic) method was about social representations of diabetes mellitus by patients. In this research, the same as ours, contaminated physical environment was mentioned as the cause of DM. Also, family and marital problems were in the social context.

At the end of interviewing, we asked them to mention their information sources. Health professionals working in primary care were most prevalent. Then, mass media programs (TV and radio) had an important role in management of patients with diabetes. Other sources with less importance were friends, relatives and other patients. Studying (the booklets and papers) only mentioned by three patients and none of them studied magazine. Almost 30 % of them said two or three origins for their source of knowledge about DM.

Dietrich (1996) found that the reaction of physician toward patients at the point of diagnosis were crucial in influencing attitudes toward perceived seriousness of the disease and its compliance. Nagelkerk et al. (2006) demonstrated positive attitude is an effective strategy that patients used by obtaining journals or books on diabetes care from multiple sources.

Stonea et al. (2005) mentioned: strong family networks and frequent family history of diabetes, could be positive in terms of providing emotional support, but might also reduce patients’ motivation to seek additional support such as offered by educational initiatives. In addition, Bangladeshis indicated a high regard for oral explanations from informal source (friends, relatives and other patients with diabetes) (Greenhalgh et al. 1998). We found these powerful family networks in our study too, so the potential for learning via oral sources is high.

Generally, in interviewees attended the Aboozar clinic with low socioeconomic status, incomplete or false information and therefore negative attitudes was more than Shariati Hospital with better condition, although their participation and freely explanation of attitudes was better than Shariati hospital.

## Strengths and Limitations of the Study

Phenomenology method has been scarcely used for exploring attitude in diabetes, so our research has been done with new style.

We used comparatively enough samples with a wide range of ages from two medical center in Tehran (capital of Iran), and the response rate for the individual interviews was high (27/30 = 90 %). According to this and also twice interviewing with different roles of a researcher (observer and participant), repeating the analysis after three-month and total analyzing, generalizability of results increased.

Triangulation strategy and then validity and reliability were done by two kinds of information (interview and direct observation) and several methods of data analysis: We have done both qualitative (Colaizzi) method and quantitative method. Informants participated in analyzing process and it repeated by peer researchers.

By using the participant’s words and because of similarities in culture of interviewer and interviewees, authenticity has been increased.

For the first time, we have explored different kinds of attitude. Sven though due to vast extension, we could not deeply overview. This research is guidance to remove the gap in educational programs that is mentioned in other articles. We did not assess any measure of diabetic control in our informants (such as glycated hemoglobin), so we were unable to relate individual perceptions or experiences to level of control, but in Alba Garcia’s research (2007) two groups were compared for blood glucose control.

## Other Issues

Knowledge has direct affect on attitude. So the patients who did not have enough knowledge about some topics, such as causes, types, correct nutrition, suitable activity and exercise, could not be an appropriate source. Furthermore, in Gillibrand and Flynn research (2001), one of the main categories emerged was “information-knowledge of illness” and participants expressed dissatisfaction with quality and quantity of information.

As Vader (2006) mentioned, belief in and commitment to the transcendental and the metaphysical, is probably the most powerful motivator of human behavior and behavior change known today. Islamic believes and activities with broad and key themes can be very useful in educational and health promotion programs. As most of the patients did not have classic symptoms of disease at the beginning of disease, in every suspected person, blood glucose testing is necessary. Many of the patients had signs and complications of disease at the time of study. These findings invite more effective programs in care and treatment of patients and changing their attitude is one of the important actions. In addition, other medical problems (for example hypertension and depression) must be treated properly.

Main objective of this study was the exploration the attitude of patients toward diabetes. We have identified a number of key themes, which may be useful in raising awareness of the experience and attitudes of patients with diabetes. Our results need consideration in designing and publicizing educational initiatives aimed for promoting patient management.
